# Association between the plasma atherogenic index and type 2 diabetes in Chinese population: prospective cohort study based on 4C study

**DOI:** 10.3389/fendo.2025.1571602

**Published:** 2025-04-16

**Authors:** Yue-Yang Zhang, Rui Cao, Qin Wan

**Affiliations:** ^1^ Department of Endocrinology and Metabolism, Affiliated Hospital of Southwest Medical University, Luzhou, China; ^2^ Metabolic Vascular Disease Key Laboratory of Sichuan Province, Luzhou, China; ^3^ Sichuan Clinical Research Center for Diabetes and Metabolism, Luzhou, China; ^4^ Sichuan Clinical Research Center for Nephropathy, Luzhou, China; ^5^ Cardiovascular and Metabolic Diseases Key Laboratory of Luzhou, Luzhou, China

**Keywords:** plasma atherogenic index, atherogenic index of plasma, type 2 diabetes, prospective cohort study, risk factor

## Abstract

**Background:**

Currently, the plasma atherogenic index (AIP) is mainly used to predict atherosclerosis and cardiovascular diseases. Therefore, we aim to investigate the potential association between AIP and type 2 diabetes through a prospective cohort study.

**Methods:**

The 4C study, a multicenter prospective cohort investigation, targets the Chinese population and initially enrolled 10,008 participants. Baseline data encompassing lifestyle, metabolic status, and various other factors were collected in 2011. A 10-year follow-up survey was subsequently conducted, ultimately including 9,092 participants. AIP, defined as the logarithmic transformation of the triglycerides to high-density lipoprotein ratio, was divided into quartiles. To explore the potential association between AIP and the risk of type 2 diabetes, Cox regression, restricted cubic spline, receiver operating characteristic curve(ROC), and subgroup analysis were employed.

**Results:**

Over a 10-year follow-up period, 693 new cases of type 2 diabetes were identified. In a fully adjusted model, AIP demonstrated a positive association with type 2 diabetes (HR: 4.40; 95% CI: 3.21, 6.04). Compared to the Q1 group, the risk of type 2 diabetes increased progressively across the Q2, Q3, and Q4 groups, with a significant trend (p-value < 0.05). Restricted cubic spline (RCS) analysis revealed an inverse L-shaped association between AIP and the risk of type 2 diabetes, with a turning point at 0.45. The ROC analysis indicates that incorporating the AIP into the base model enhances its diagnostic performance for type 2 diabetes. Furthermore, similar patterns were observed in the subgroup analyses.

**Conclusions:**

Among the Chinese population, elevated AIP levels are positively correlated with an increased risk of type 2 diabetes, indicating that AIP could potentially serve as a biomarker for assessing the risk of developing type 2 diabetes.

## Highlights

Among the Chinese population, elevated AIP levels are positively correlated with an increased risk of type 2 diabetes, indicating that AIP could potentially serve as a biomarker for assessing the risk of developing type 2 diabetes.

More and more people worldwide are suffering from type 2 diabetes. Although other studies have found an association between AIP and type 2 diabetes, there is still a lack of studies exploring the association between AIP and type 2 diabetes in a large cohort in the Chinese population.

## Introduction

Type 2 diabetes is a critical chronic condition marked by elevated blood glucose levels resulting from either relative or absolute insulin insufficiency (1, 2). The 2019 Global Burden of Disease, Injuries, and Risk Factors study identified diabetes as the eighth leading risk factor for both mortality and disability, with an estimated 460 million individuals globally affected by the disease in 2019 (3). The 2021 edition of the International Diabetes Federation’s (IDF) Diabetes Atlas reported that the number of people living with diabetes had risen to 537 million. Projections indicate that this number could escalate to 643 million by 2030 and reach an alarming 783 million by 2045, potentially leading to global healthcare costs surpassing 1,054 billion USD (4). These statistics emphasize the profound impact of diabetes on global public health and highlight the urgent need for effective early detection strategies for type 2 diabetes across the general population (5, 6).

Recent advancements in high-throughput detection technologies have led to the identification of several significant biomarkers for predicting type 2 diabetes, including plasma lipid profiles, amino acids, and gut microbiota (7). Plasma lipid profiles, encompassing triglycerides (TG), total cholesterol (TC), high-density lipoprotein cholesterol (HDL-C), and low-density lipoprotein cholesterol (LDL-C), are recognized as crucial risk factors and predictive markers for metabolic disorders (8). The plasma atherogenic index (AIP), introduced by Dobiásová et al. (9) in 2001, serves as a predictive marker for atherosclerosis and is derived from the logarithmic transformation of the TG-to-HDL-C molar ratio. AIP is positively correlated with cholesterol esterification rates, lipoprotein particle size, and residual lipoprotein levels, and is therefore utilized in identifying atherogenic dyslipidemia (10, 11). Extensive research has validated the reliability of AIP in predicting atherosclerosis and cardiovascular diseases, underscoring its potential as a valuable biomarker (12–14).

Furthermore, a cross-sectional study derived from the National Health and Nutrition Examination Survey (NHANES) has identified a nonlinear relationship between AIP and insulin resistance (15). Despite this, research focusing on the association between AIP and type 2 diabetes remains limited, with the majority of studies being cross-sectional in nature. To address this gap, we undertook a prospective cohort study utilizing the Chinese Heart Disease and Cancer Cohort (4C) to more comprehensively investigate the nonlinear association between AIP and type 2 diabetes within the general Chinese population (16).

## Method

### Study design and population

The 4C study is a comprehensive, multicenter, population-based prospective cohort investigation designed to elucidate the relationships between metabolic factors and specific clinical outcomes, with a particular focus on diabetes and major cardiovascular events. The study protocol, along with the informed consent documentation, received approval from the Human Research Committee of Ruijin Hospital, Shanghai Jiao Tong University School of Medicine, China. All participants provided informed written consent prior to their involvement (17).

The 4C study encompasses 20 community research sites across 16 provinces, autonomous regions, and municipalities throughout mainland China. Initially, eligible men and women aged 40 and older were identified through the resident registration systems of each research site. Trained community health workers then visited the homes of these individuals, invited them to participate in the study, and managed subsequent follow-ups. The research population for this study originates from the 4C project conducted in five cities in southwestern China. Between 2010 and 2011, baseline assessments were carried out on 10,008 participants, including face-to-face interviews, physical examinations, standard oral glucose tolerance tests (OGTT), and blood sample collection. Follow-up surveys were performed in 2014, 2016, and 2021, resulting in a retained cohort of 9,126 participants and a follow-up rate of 91.19%.

**Figure 1:**
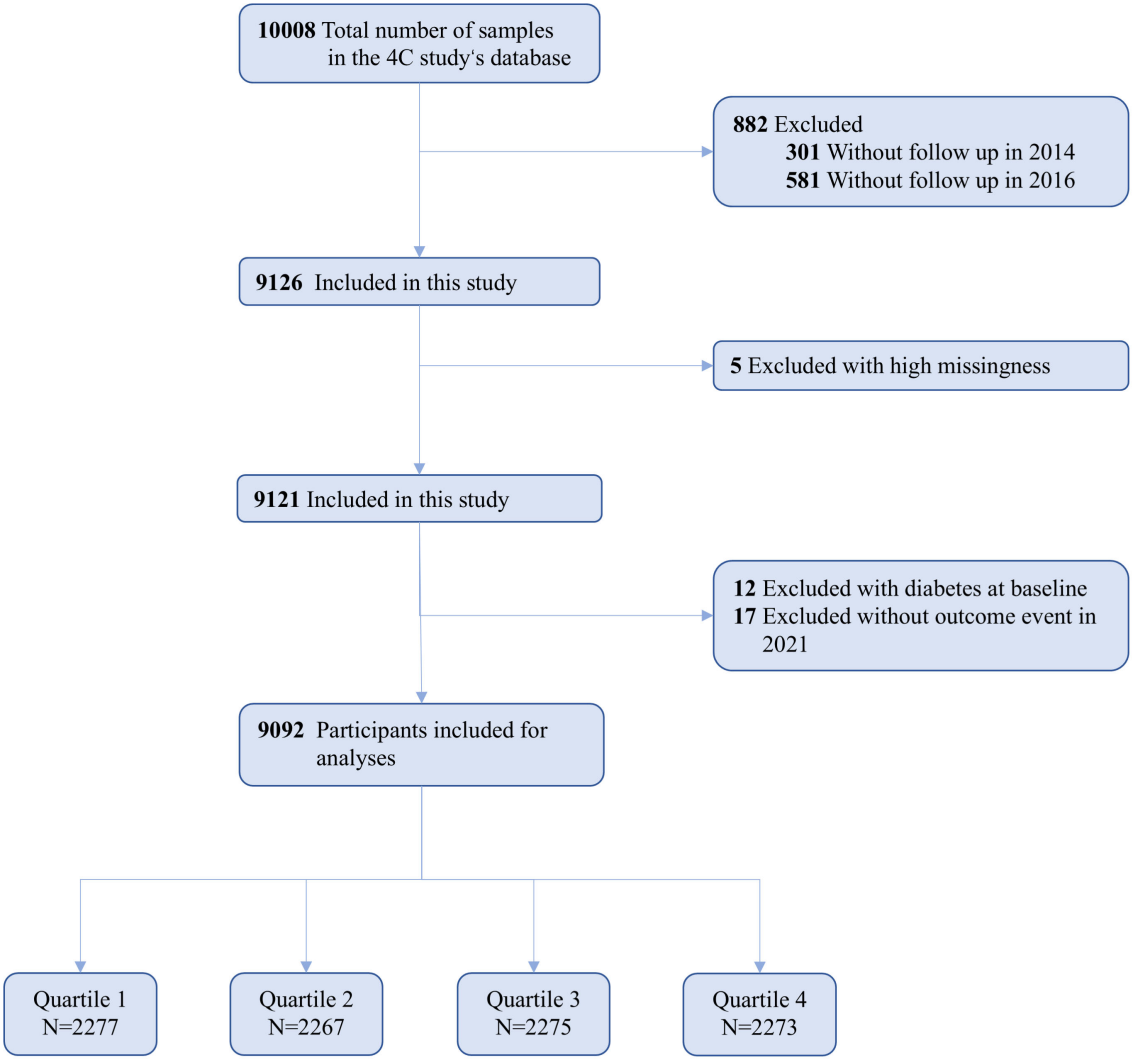
Flowchart for the selection of the analyzed study sample from the 4C study’s database.

Initially, we excluded 5 participants due to missing crucial baseline information, such as sex and age. Subsequently, 12 participants were excluded because they had been previously diagnosed with type 2 diabetes, and an additional 17 participants were removed due to incomplete outcome event data during follow-up. Consequently, the final analysis included 9,092 participants (**Figure 1**). The baseline diagnosis of type 2 diabetes mellitus is initially determined based on self-reported information and medical records. This is subsequently validated by professionals using fasting blood glucose levels, HbA1c, and OGTT results.

### Data collection

Baseline data were systematically collected each morning at multiple community hospitals. Participants were instructed to fast for a minimum of 10 hours prior to their appointment. Trained research staff utilized standardized questionnaires to gather detailed demographic information, dietary habits, lifestyle factors, medical history, and medication usage. Professional nurses, adhering to standardized procedures, measured participants’ weight, height, blood pressure, and waist circumference. Prior to these measurements, participants were required to rest quietly for at least 30 minutes and abstain from alcohol, smoking, tea, and physical exercise. Blood pressure was recorded using an electronic sphygmomanometer (HEM-752 FUZZY; Omron, Dalian, China), with three separate readings taken and averaged for accuracy. Blood samples were subsequently collected and sent to a centralized laboratory for analysis of HbA1c levels and circulating metabolites, including lipids. Glycated hemoglobin in capillary whole blood was quantified using high-performance liquid chromatography (VARIANT II system; Bio-Rad, Hercules, CA), and lipid levels were measured using an automated analyzer (Abbott Laboratories, Abbott Park, IL). Smoking status was categorized as current smokers (defined as those smoking at least 7 cigarettes per week for a minimum of 6 months) and former smokers (those who had previously smoked at least 7 cigarettes per week for 6 months but were not currently smoking). Drinking status was classified as current drinkers (participants consuming alcohol at least once a week for a minimum of 6 months) and former drinkers (those who had consumed alcohol at least once a week for 6 months but were no longer drinking) (18). AIP was calculated as the logarithmic transformation of the ratio of TG to HDL-C (19). Participants were then classified into four quartile groups based on AIP values: Quartile 1 (Q1), AIP < -0.15; Quartile 2 (Q2), AIP ≥ -0.155 and < 0.025; Quartile 3 (Q3), AIP ≥ 0.025 and < 0.228; Quartile 4 (Q4), AIP ≥ 0.228.

### Ascertainment of covariates

In line with prior research, we incorporated a range of potential confounding variables into the baseline statistical analyses (20). These variables include demographic factors such as age and sex (classified as “male” and “female”), educational attainment (categorized as “below high school” and “high school or above”), as well as lifestyle factors including current smoking status (“yes” or “no”) and current drinking status (“yes” or “no”). Additionally, we adjusted for various clinical measures: systolic blood pressure (SBP), diastolic blood pressure (DBP), body mass index (BMI), fasting blood glucose (FBG), TG, serum creatinine (Cr), TC, HDL-C, LDL-C, and glycated hemoglobin (HbA1c).

### Assessment of type 2 diabetes

To ascertain disease status, we established comprehensive linkages with hospital databases, national insurance system medical records, and resident records to facilitate thorough information collection. Throughout the follow-up period, type 2 diabetes diagnosis adhered to the 2013 American Diabetes Association criteria, necessitating fulfillment of at least one of the following diagnostic criteria: 1. Fasting blood glucose level of 7.0 mmol/L or higher; 2. Postprandial blood glucose level of 11.1 mmol/L or higher at 2 hours during an oral glucose tolerance test (OGTT); 3. Glycated hemoglobin level of 6.5% (48 mmol/mol) or above; 4. Clinical diagnosis confirmed by a licensed physician.

### Statistical analysis

The baseline characteristics of participants were systematically described using appropriate statistical methods tailored to the data type. Continuous variables were expressed as means with standard deviations, while categorical variables were reported as counts with percentages. Normality of data was assessed using analysis of variance (ANOVA) for normally distributed variables, and the Kruskal-Wallis test was employed for skewed data distributions. Categorical variables were compared using chi-square tests. Kaplan-Meier curves were generated to estimate the cumulative incidence of type 2 diabetes, with group differences assessed using the log-rank test. Additionally, the absence of crossover between curves suggests that the proportional hazards assumption was met. The association between the AIP and type 2 diabetes was evaluated using Cox proportional hazards regression, employing four distinct models to present hazard ratios (HR) with 95% confidence intervals (CI). Model 1 was an unadjusted model. Model 2 adjusted for age and sex. Model 3 included adjustments for age, sex, BMI, DBP, SBP, LDL-C, TC, Cr, FBG, and HbA1c. Model 4 was fully adjusted for age, sex, BMI, DBP, SBP, LDL-C, TC, Cr, FBG, HbA1c, smoking status, alcohol consumption, and education level. Trend p-values were calculated by treating AIP quartiles as an ordinal variable. Furthermore, a 5-knot restricted cubic spline (RCS) analysis was performed to explore the nonlinear relationship between AIP and type 2 diabetes, adjusting for Model 4 and dichotomizing continuous variables at the median (21). Existing studies generally recommend using 3–5 RCS nodes. Given the large sample size of this study, we selected a 5-node RCS for analysis. The predictive value of AIP for type 2 diabetes risk was also evaluated using receiver operating characteristic (ROC) curve analysis. Subgroup analyses were conducted to investigate interactions with categorical confounding variables such as age (<60, ≥60), sex (male, female), BMI (<24, ≥24), and history of hypertension(yes, no) utilizing Cox regression. Non-significant interaction p-values indicated consistency across strata, while significant p-values suggested potential subgroup-specific effects.

All statistical analyses were executed using SPSS version 26.0 and R version 4.3.3, with forest plots generated using GraphPad Prism version 10.0. A two-sided p-value of less than 0.05 was deemed statistically significant.

## Result

### Baseline characteristics

**Supplementary Table S1** presents the baseline characteristics of both missing respondents and non-missing respondents, demonstrating no significant differences between the two groups. The study cohort comprised 9,092 participants, with a mean age of 58.50 ± 10.02 years, of whom 33.9% were male. Among the participants, 1,308 individuals (14.4%) were identified as current smokers, 1,308 (27.7%) were regular drinkers, and 2,689 (37.9%) had achieved at least a high school education. **Table 1** presents the baseline characteristics of participants stratified by AIP quartiles. Individuals in higher AIP quartiles were notably older on average and exhibited a higher prevalence of male gender and smoking habits. Furthermore, those in higher AIP quartiles had elevated levels of BMI, SBP, DBP, TC, TG, Cr, FBG, and HbA1c, while their HDL-C levels were significantly lower.

**Table 1:** Baseline characteristics of participants by AIP quartile.

Variables	Total	Q1 (<-0.15)	Q2 (-0.15-0.03)	Q3 (0.03-0.23)	Q4 (>0.23)	P
N	9092	2277	2267	2275	2273	–
Age, years	58.50 ± 10.02	56.83 ± 10.19	58.61 ± 10.18	59.17 ± 9.87	59.39 ± 9.61	<0.01
Male (%)	3085 (33.9)	686 (30.1)	739 (32.6)	790 (34.7)	870 (38.3)	<0.01
BMI, kg/m^2^	23.91 ± 3.35	22.24 ± 3.08	23.56 ± 3.24	24.52 ± 3.17	25.29 ± 3.13	<0.01
DBP, mmHg	77.62 ± 11.54	74.24 ± 10.92	76.91 ± 11.40	78.99 ± 11.56	80.40 ± 11.28	<0.01
SBP, mmHg	127.40 ± 20.92	121.35 ± 20.31	125.89 ± 20.68	130.10 ± 20.72	132.15 ± 20.29	<0.01
LDL-c, mmol/L	2.59 ± 0.82	2.44 ± 0.75	2.61 ± 0.84	2.73 ± 0.86	2.55 ± 0.82	<0.01
HDL-c, mmol/L	1.24 ± 0.34	1.53 ± 0.34	1.29 ± 0.29	1.16 ± 0.26	1.00 ± 0.22	<0.01
TC, mmol/L	4.59 ± 1.13	4.48 ± 1.03	4.48 ± 1.12	4.61 ± 1.14	4.77 ± 1.18	<0.01
TG, mmol/L	1.62 ± 1.28	0.79 ± 0.19	1.11 ± 0.27	1.54 ± 0.37	3.04 ± 1.81	<0.01
Cr, μmol/L	65.10 ± 21.80	63.09 ± 16.29	64.18 ± 27.23	65.24 ± 18.83	67.92 ± 22.98	<0.01
FBG, mmol/L	5.92 ± 1.68	5.50 ± 1.20	5.73 ± 1.46	6.01 ± 1.71	6.41 ± 2.05	<0.01
HbA1c, %	6.16 ± 1.10	5.89 ± 0.84	6.05 ± 0.97	6.22 ± 1.15	6.45 ± 1.32	<0.01
Current smoker	1308 (14.4)	287 (12.6)	301 (13.3)	330 (14.5)	390 (17.2)	<0.01
Current drinker	2515 (27.7)	679 (29.8)	597 (26.3)	615 (27.0)	624 (27.5)	0.06
Senior high school and above	2689 (29.5)	663 (29.1)	677 (29.9)	701 (30.8)	648 (28.5)	0.38

Data are summarized as number (percentage), mean ± standard deviation. BMI, body mass index; DBP, diastolic blood pressure; SBP, systolic blood pressure; LDL-C, low-density lipoprotein cholesterol; HDL-C, high-density lipoprotein cholesterol; TC, total cholesterol; TG, triglyceride; Cr, creatinine; FBG, fasting blood glucose; HbA1c, glycated hemoglobin.

### Association of AIP with type 2 diabetes

**Figure 2:**
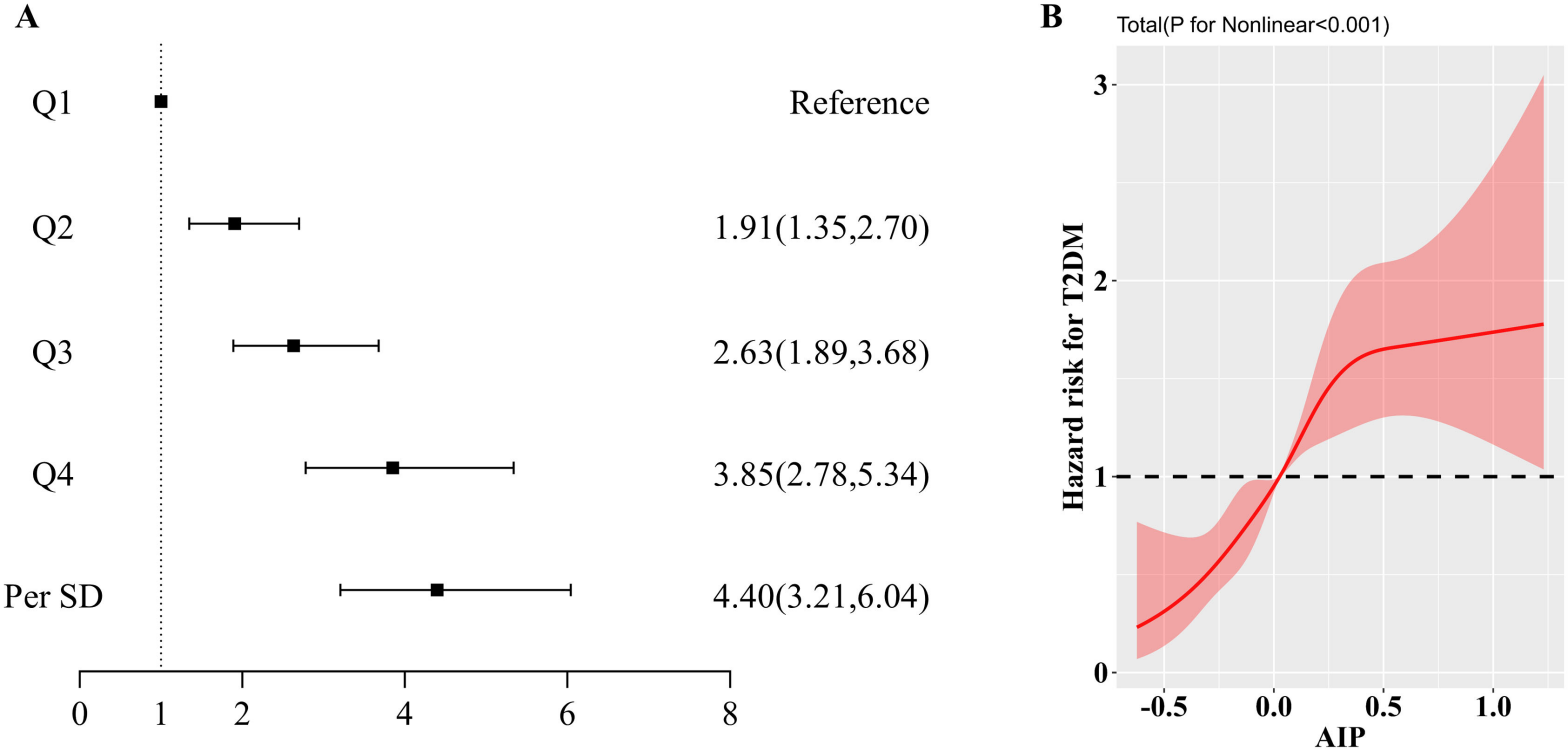
**(A)**. Forest plot of the association between AIP and type 2 diabetes mellitus; **(B)**. Results of RCS analysis of the association between AIP and type 2 diabetes mellitus. Adjusted for age, sex, BMI, DBP, SBP, LDL-C, TC, Cr, FBG, HbA1c, smoking status, alcohol consumption, and education level.

As shown in **Supplementary Figure S1**, the Kaplan-Meier survival curves illustrate a higher cumulative incidence of type 2 diabetes among participants with elevated AIP. Additionally, the absence of crossover between the group curves suggests compliance with the proportional hazards assumption. The outcomes of the Cox regression analysis are detailed in **Table 2** and illustrated in **Figure 2A**. Over the course of the 10-year follow-up, a total of 693 new cases of type 2 diabetes were documented, yielding an overall incidence rate of 7.6%. The incidence rates for type 2 diabetes across AIP quartiles were 2.46% for Quartile 1 (Q1), 5.43% for Quartile 2 (Q2), 8.97% for Quartile 3 (Q3), and 13.6% for Quartile 4 (Q4). In the fully adjusted Model 4, AIP quartiles demonstrated a statistically significant association with the incidence of type 2 diabetes. Specifically, relative to Q1, the HR for Q2, Q3, and Q4 were 1.91 (95% CI: 1.35, 2.70), 2.63 (95% CI: 1.89, 3.68), and 3.85 (95% CI: 2.78, 5.34), respectively (P for trend < 0.01). Treating AIP as a continuous variable, each standard deviation increase in AIP was linked to an elevated risk of type 2 diabetes, with an HR of 4.40 (95% CI: 3.21, 6.04). As depicted in **Figure 2B**, the restricted cubic spline analysis revealed a nonlinear and reverse L-shaped relationship between continuous AIP and the risk of type 2 diabetes. Threshold effect analysis identified a critical cutoff value of 0.45, below which there was a rapid increase in the prevalence of type 2 diabetes (P for Nonlinear < 0.01). In addition, as shown in **Supplementary Figure S2**, incorporating AIP significantly enhanced the predictive efficacy of the base model for type 2 diabetes (AUC: 0.74 vs. 0.69).

**Table 2:** Hazard ratios and 95% CIs for the association of AIP with type 2 diabetes.

Variables	Q1	Q2	Q3	Q4	Per SD	P for trend
Event/total	56/2277	123/2267	204/2275	310/2273	693/9092	
Incident rate (%)	2.46	5.43	8.97	13.6	7.6	
Model 1	Reference	2.23 (1.63,3.06)	3.76 (2.80,5.05)	5.89 (4.43,7.82)	5.20 (4.20,6.43)	<0.01
Model 2	Reference	2.19 (1.60,3.01)	3.66 (2.71,4.92)	5.77 (4.34,7.68)	5.25 (4.23,6.51)	<0.01
Model 3	Reference	1.84 (1.32,2.55)	2.57 (1.88,3.51)	3.71 (2.73,5.02)	4.32 (3.20,5.82)	<0.01
Model 4	Reference	1.91 (1.35,2.70)	2.63 (1.89,3.68)	3.85 (2.78,5.34)	4.40 (3.21,6.04)	<0.01

Model 1: unadjusted model;

Model 2: adjusted for age and sex;

Model 3: adjusted for age, sex, BMI, DBP, SBP, LDL-C, TC, Cr, FBG, and HbA1c;

Model 4: adjusted for age, sex, BMI, DBP, SBP, LDL-C, TC, Cr, FBG, HbA1c, smoking status, alcohol consumption, and education level.

AIP, Atherogenic Index of Plasma; Per SD, hazard ratio for per SD change in AIP.

### Subgroup analyses

To gain deeper insights into the relationship between AIP and type 2 diabetes, we performed subgroup analyses stratified by age, sex, BMI, and history of hypertension as detailed in **Table 3**. In each subgroup, an increase of 1 standard deviation in AIP consistently correlated with a heightened risk of developing type 2 diabetes. Additionally, when AIP was categorized into quartiles, the observed associations remained robust, and the nonlinear relationship between AIP and type 2 diabetes persisted. Notably, no significant interactions were detected between AIP levels and the subgroup variables (P for interaction > 0.05).

**Table 3:** Subgroup analyses for the association of the AIP with type 2 diabetes.

Variables	Q1	Q2	Q3	Q4	Per SD	P for interaction
Age						
<60 years	Reference	1.52 (1.22,2.42)	2.46 (1.60,3.79)	3.71 (2.43,5.65)	4.32 (2.82,6.63)	0.46
≥60 years	Reference	2.09 (1.31,3.33)	2.56 (1.63,4.03)	3.50 (2.25,5.45)	4.06 (2.66,6.18)	
BMI						
<24 kg/m^2^	Reference	1.75 (1.07,2.86)	2.12 (1.30,3.45)	4.41 (2.78,7.01)	6.84 (3.99,11.72)	0.28
≥24 kg/m^2^	Reference	1.86 (1.12,3.07)	2.60 (1.61,4.18)	3.33 (2.08,5.32)	3.21 (2.15,4.80)	
Sex						
Male	Reference	2.30 (1.15,4.57)	3.32 (1.72,6.42)	4.31 (2.25,8.24)	4.15 (2.37,7.27)	0.22
Female	Reference	1.78 (1.19,2.67)	2.39 (1.62,3.52)	3.74 (2.56,4.45)	4.48 (3.06,6.57)	
Hypertensive						
Yes	Reference	1.89 (1.36,2.68)	2.56 (1.83,3.58)	3.53 (2.48,5.03)	7.65 (4.42,13.26)	0.34
No	Reference	1.66 (1.10,2.50)	2.29 (1.54,3.40)	3.20 (2.08,4.92)	7.64 (4.76,13.53)	

Adjusted for age, sex, BMI, DBP, SBP, LDL-C, TC, Cr, FBG, HbA1c, smoking status, alcohol consumption, and education level. BMI, body mass index; AIP, Atherogenic Index of Plasma; Per SD, hazard ratio for per SD change in AIP.

## Discussion

In this prospective cohort study encompassing 9,092 Chinese adults, we identified a persistent positive association between plasma AIP levels and the risk of type 2 diabetes, even after comprehensive adjustment for various confounding factors. The robustness of this association was further corroborated by subgroup analyses, underscoring its reliability across different demographic segments. Furthermore, restricted cubic spline (RCS) analysis revealed a nonlinear relationship between AIP and type 2 diabetes risk, manifested as a reverse L-shaped curve with a critical threshold at 0.45. These findings underscore the potential of AIP as a significant early biomarker for predicting type 2 diabetes.

Type 2 diabetes is a chronic condition characterized by a gradual progression over an individual’s lifetime, with an alarming trend of increased diagnoses among younger populations. Once established, type 2 diabetes becomes a lifelong affliction, profoundly impacting quality of life through a range of complications, including both microvascular and macrovascular issues, and imposing a significant economic burden on individuals and healthcare systems alike (22, 23). Current research underscores that proactive prevention and treatment strategies can markedly mitigate diabetes-related complications and curtail associated healthcare costs (24). Evidence suggests that lifestyle interventions, such as dietary modifications and enhanced physical activity, may surpass pharmacological treatments in delaying disease progression and aiding early prevention (25). Presently, diabetes biomarkers predominantly focus on indicators related to glucose metabolism. Despite their efficacy, these biomarkers often fall short in predicting and diagnosing type 2 diabetes beyond conventional metrics like fasting blood glucose (26). Thus, identifying novel biomarkers for early prediction of type 2 diabetes remains a crucial objective.

While some studies have identified associations between individual plasma lipid components, such as LDL cholesterol, and type 2 diabetes, these components alone may not provide a comprehensive assessment of diabetes risk (27). Consequently, researchers have increasingly focused on composite indicators, such as the glucose-triglyceride index, to better evaluate their relationship with type 2 diabetes (28). In contrast, the AIP offers a more integrated approach by utilizing plasma lipid profiles. AIP is associated with lipoprotein particle size and reflects the interplay between anti-atherogenic and pro-atherogenic particles. The National Cholesterol Education Program recognizes AIP as a significant marker of plasma atherosclerosis and a dependable predictor of cardiovascular risk (29). A longitudinal study involving 8,760 participants from the China Health and Retirement Longitudinal Study(CHARLS) demonstrated that variations in AIP from baseline to follow-up were predictive of type 2 diabetes risk. Specifically, individuals with persistently high AIP or those experiencing shifts from high to low or low to high AIP had about a 1.5-fold increased risk of type 2 diabetes compared to those with consistently low AIP levels (30). An earlier meta-analysis also affirmed that AIP serves as a straightforward and reliable marker for assessing type 2 diabetes risk (31). Furthermore, an analysis of NHANES data corroborated our findings, revealing a reverse L-shaped association between AIP and type 2 diabetes risk, with a notable breakpoint at 0.45 (15). Another study from CHARLS also found an association between AIP and the risk of type 2 diabetes in a Chinese population. However, it is worth noting that the threshold for AIP identified in this study was -0.04 (32). We hypothesize that this discrepancy may arise from differences in the populations involved in the CHARLS and 4C studies. Therefore, future cohort studies with larger sample sizes and broader geographic distributions will be essential to further refine and clarify this issue.

We hypothesize that the association between AIP and type 2 diabetes may be explained through several potential mechanisms. Firstly, AIP is a marker of plasma lipoprotein metabolism and exhibits a positive correlation with small dense LDL (sdLDL). SdLDL is known to be a predictor of atherosclerosis due to its small size, low plasma clearance rate, and heightened sensitivity to oxidative stress, which can precipitate inflammation in the subendothelial space (33, 34). Existing studies have demonstrated that AIP is the biomarker that best reflects changes in sdLDL, which, in turn, serves as a significant predictor of the onset of diabetes mellitus (35, 36). Additionally, the production of sdLDL is primarily influenced by TG metabolism. In the state of insulin resistance, ApoB degradation is inhibited, leading to an overproduction of very low-density lipoproteins (VLDL), which results in the predominance of sdLDL due to increased activity of cholesteryl ester transfer protein and hepatic lipases (37, 38). Haffner et al. (39) have also long identified that sdLDL is significantly associated with selective pancreatic β-cell defects and insulin resistance. Furthermore, emerging research highlights inflammation as a crucial pathogenic factor in type 2 diabetes (40, 41). It is proposed that type 2 diabetes represents the culmination of an acute-phase response characterized by substantial cytokine release from adipose tissue and macrophages, which exacerbates cellular dysfunction. This inflammatory process necessitates considerable energy expenditure and is modulated by genetic factors (42). Nevertheless, the precise mechanisms through which AIP influences type 2 diabetes remain unclear and warrant further investigation through both basic and clinical research. It is important to note that although there was no statistical difference in the percentage of drinkers among the four groups of participants in this study, the effect of alcohol consumption on lipid metabolism is intrinsic. Alcohol consumption can influence lipid metabolism by altering the activity of key proteins and enzymes, such as cholesteryl ester transfer protein and hepatic lipase. Furthermore, the impact of lipoproteins on vascular wall cells can be regulated by ethanol (43).

### Strengths and limitations

This study possesses several notable strengths. Firstly, it features a large sample size, which is comparatively larger than that of other studies examining the association between AIP and type 2 diabetes within the Chinese population, thereby offering a more comprehensive representation of the general Chinese demographic. Secondly, the prospective nature of the cohort study, coupled with an extended follow-up period and a high response rate, provides a robust framework for minimizing biases inherent in cross-sectional studies and enhances the reliability of the findings. Thirdly, leveraging data from the 4C study, the collection of data and diagnosis of diseases were carried out by trained professionals adhering to standardized protocols, which substantially mitigates potential biases stemming from human factors. Finally, the application of RCS and stratified analyses has bolstered the statistical power and affirmed the robustness of the results.

Nevertheless, this study does have several limitations. Firstly, variables that were not accounted for, such as dietary patterns, ethnic differences, and lifestyle factors, could also influence AIP levels. Therefore, we will refine the content in subsequent follow-up studies to enhance the reliability of the results. Secondly, since the study primarily involves participants from China, extrapolating the findings to other populations should be approached with caution. Despite these limitations, the robust cohort established in this study significantly strengthens the reliability and validity of the results. Finally, due to the inherent limitations of observational studies, we were unable to establish definitive causal associations. Therefore, further follow-up is necessary to explore the mechanisms by which AIP affects the risk of type 2 diabetes mellitus, including the development of animal and cellular models.

## Conclusions

In summary, our study reveals that AIP exhibits a reverse L-shaped relationship with the risk of type 2 diabetes, with a threshold value identified at 0.45. Elevated AIP levels are significantly correlated with an increased risk of developing type 2 diabetes. These results highlight the importance of ongoing monitoring and the maintenance of lower AIP levels as potential strategies for the early detection and prevention of type 2 diabetes. Clinicians, particularly those in community settings, can help prevent type 2 diabetes by focusing on an individual’s AIP early on and using this information to develop more personalized treatment strategies. For example, physicians may choose appropriate lipid-lowering drugs to improve a patient’s elevated AIP. It is important to note that clinicians should assess changes in a patient’s AIP when evaluating the efficacy of a drug for hyperlipidemia, rather than focusing on TG and TC alone.

## Data Availability

The raw data supporting the conclusions of this article will be made available by the authors, without undue reservation.

## References

[1] Chan JCN Lim LL Wareham NJ Shaw JE Orchard TJ Zhang P. The Lancet Commission on diabetes: using data to transform diabetes care and patient lives. Lancet Lond Engl. (2021) 396. doi: 10.1016/S0140-6736(20)32374-6 33189186

[2] Chatterjee S Khunti K Davies MJ. Type 2 diabetes. Lancet Lond Engl. (2017) 389. doi: 10.1016/S0140-6736(17)30058-2 28190580

[3] GBD 2021 Diabetes Collaborators. Global, regional, and national burden of diabetes from 1990 to 2021, with projections of prevalence to 2050: a systematic analysis for the Global Burden of Disease Study 2021. Lancet Lond Engl. (2023) 402:203. doi: 10.1016/S0140-6736(23)01301-6 PMC10364581 37356446

[4] Gregory GA Robinson TIG Linklater SE Wang F Colagiuri S de Beaufort C. Global incidence, prevalence, and mortality of type 1 diabetes in 2021 with projection to 2040: a modelling study. Lancet Diabetes Endocrinol. (2022) 10. doi: 10.1016/S2213-8587(22)00218-2 36113507

[5] Sun H Saeedi P Karuranga S Pinkepank M Ogurtsova K Duncan BB. IDF Diabetes Atlas: Global, regional and country-level diabetes prevalence estimates for 2021 and projections for 2045. Diabetes Res Clin Pract. (2022) 183. doi: 10.1016/j.diabres.2021.109119 PMC11057359 34879977

[6] Liu Y Yao S Shan X Luo Y Yang L Dai W. Time trends and advances in the management of global, regional, and national diabetes in adolescents and young adults aged 10-24 years, 1990-2021: analysis for the global burden of disease study 2021. Diabetol Metab Syndr. (2024) 16. doi: 10.1186/s13098-024-01491-w PMC11515246 39456070

[7] Laakso M. Biomarkers for type 2 diabetes. Mol Metab. (2019) 27S. doi: 10.1016/j.molmet.2019.06.016 PMC6768493 31500825

[8] Sandesara PB Virani SS Fazio S Shapiro MD. The forgotten lipids: triglycerides, remnant cholesterol, and atherosclerotic cardiovascular disease risk. Endocr Rev. (2019) 40:537–57. doi: 10.1210/er.2018-00184 PMC6416708 30312399

[9] Dobiásová M Frohlich J. The plasma parameter log (TG/HDL-C) as an atherogenic index: correlation with lipoprotein particle size and esterification rate in apoB-lipoprotein-depleted plasma (FER(HDL)). Clin Biochem. (2001) 34. doi: 10.1016/s0009-9120(01)00263-6 11738396

[10] Dobiásová M. Atherogenic index of plasma [log(triglycerides/HDL-cholesterol)]: theoretical and practical implications. Clin Chem. (2004) 50. doi: 10.1373/clinchem.2004.033175 15229146

[11] Fernández-Macías JC Ochoa-Martínez AC Varela-Silva JA Pérez-Maldonado IN. Atherogenic index of plasma: novel predictive biomarker for cardiovascular illnesses. Arch Med Res. (2019) 50:285–94. doi: 10.1016/j.arcmed.2019.08.009 31593853

[12] Fu L Zhou Y Sun J Zhu Z Xing Z Zhou S. Atherogenic index of plasma is associated with major adverse cardiovascular events in patients with type 2 diabetes mellitus. Cardiovasc Diabetol. (2021) 20. doi: 10.1186/s12933-021-01393-5 PMC8493717 34610830

[13] Zheng Y Li C Yang J Seery S Qi Y Wang W. Atherogenic index of plasma for non-diabetic, coronary artery disease patients after percutaneous coronary intervention: a prospective study of the long-term outcomes in China. Cardiovasc Diabetol. (2022) 21. doi: 10.1186/s12933-022-01459-y PMC8864872 35193553

[14] Ismaiel A Ciobanu OS Ismaiel M Leucuta DC Popa SL David L. Atherogenic index of plasma in non-alcoholic fatty liver disease: systematic review and meta-analysis. Biomedicines. (2022) 10. doi: 10.3390/biomedicines10092101 PMC9495578 36140201

[15] Yin B Wu Z Xia Y Xiao S Chen L Li Y. Non-linear association of atherogenic index of plasma with insulin resistance and type 2 diabetes: a cross-sectional study. Cardiovasc Diabetol. (2023) 22:157. doi: 10.1186/s12933-023-01886-5 37386500 PMC10311747

[16] Lu J Wang S Li M Gao Z Xu Y Zhao X. Association of serum bile acids profile and pathway dysregulation with the risk of developing diabetes among normoglycemic Chinese adults: findings from the 4C study. Diabetes Care. (2021) 44:499–510. doi: 10.2337/dc20-0884 33355246

[17] Lu J He J Li M Tang X Hu R Shi L. Predictive value of fasting glucose, postload glucose, and hemoglobin A1c on risk of diabetes and complications in Chinese adults. Diabetes Care. (2019) 42(11):1539–48. doi: 10.2337/dc18-1390 31152120

[18] Wang T Lu J Su Q Chen Y Bi Y Mu Y. Ideal cardiovascular health metrics and major cardiovascular events in patients with prediabetes and diabetes. JAMA Cardiol. (2019) 4. doi: 10.1001/jamacardio.2019.2499 PMC6669896 31365039

[19] Zhu Y Zhang JL Yan XJ Sun L Ji Y Wang FF. Effect of dapagliflozin on the prognosis of patients with acute myocardial infarction undergoing percutaneous coronary intervention. Cardiovasc Diabetol. (2022) 21. doi: 10.1186/s12933-022-01627-0 PMC9482258 36114538

[20] Warren B Pankow JS Matsushita K Punjabi NM Daya NR Grams M. Comparative prognostic performance of definitions of prediabetes: a prospective cohort analysis of the Atherosclerosis Risk in Communities (ARIC) study. Lancet Diabetes Endocrinol. (2017) 5. doi: 10.1016/S2213-8587(16)30321-7 PMC5183486 27863979

[21] Dou J Guo C Wang Y Peng Z Wu R Li Q. Association between triglyceride glucose-body mass and one-year all-cause mortality of patients with heart failure: a retrospective study utilizing the MIMIC-IV database. Cardiovasc Diabetol. (2023) 22. doi: 10.1186/s12933-023-02047-4 PMC10634170 37940979

[22] Zhang P Zhang X Brown J Vistisen D Sicree R Shaw J. Global healthcare expenditure on diabetes for 2010 and 2030. Diabetes Res Clin Pract. (2010) 87. doi: 10.1016/j.diabres.2010.01.026 20171754

[23] Litwak L Goh SY Hussein Z Malek R Prusty V Khamseh ME. Prevalence of diabetes complications in people with type 2 diabetes mellitus and its association with baseline characteristics in the multinational A1chieve study. Diabetol Metab Syndr. (2013) 5. doi: 10.1186/1758-5996-5-57 PMC3854020 24228724

[24] Chen ZZ Gerszten RE. Metabolomics and proteomics in type 2 diabetes. Circ Res. (2020) 126:1613. doi: 10.1161/CIRCRESAHA.120.315898 32437301 PMC11118076

[25] Knowler WC Barrett-Connor E Fowler SE Hamman RF Lachin JM Walker EA. Reduction in the incidence of type 2 diabetes with lifestyle intervention or metformin. N Engl J Med. (2002) 346. doi: 10.1056/NEJMoa012512 PMC1370926 11832527

[26] Hu X Chen S Ye S Chen W Zhou Y. New insights into the role of immunity and inflammation in diabetic kidney disease in the omics era. Front Immunol. (2024) 15:1342837. doi: 10.3389/fimmu.2024.1342837 38487541 PMC10937589

[27] Swerdlow DI Preiss D Kuchenbaecker KB Holmes MV Engmann JE Shah T. HMG-coenzyme a reductase inhibition, type 2 diabetes, and bodyweight: evidence from genetic analysis and randomised trials. Lancet Lond Engl. (2015) 385. doi: 10.1016/S0140-6736(14)61183-1 PMC4322187 25262344

[28] Yao Y Wang B Geng T Chen J Chen W Li L. The association between TyG and all-cause/non-cardiovascular mortality in general patients with type 2 diabetes mellitus is modified by age: results from the cohort study of NHANES 1999-2018. Cardiovasc Diabetol. (2024) 23. doi: 10.1186/s12933-024-02120-6 PMC10823741 38281973

[29] Third Report of the National Cholesterol Education Program (NCEP) Expert Panel on Detection, Evaluation, and Treatment of High Blood Cholesterol in Adults (Adult Treatment Panel III) final report. Circulation. (2002) 106. https://pubmed.ncbi.nlm.nih.gov/12485966/ Accessed August 2, 2024. 12485966

[30] Yi Q Ren Z Bai G Zhu S Li S Li C. The longitudinal effect of the atherogenic index of plasma on type 2 diabetes in middle-aged and older Chinese. Acta Diabetol. (2022) 59. doi: 10.1007/s00592-021-01801-y 34648090

[31] Zhu XW Deng FY Lei SF. Meta-analysis of Atherogenic Index of Plasma and other lipid parameters in relation to risk of type 2 diabetes mellitus. Prim Care Diabetes. (2015) 9:60–7. doi: 10.1016/j.pcd.2014.03.007 24810146

[32] Jiang L Li L Xu Z Tang Y Zhai Y Fu X. Non-linear associations of atherogenic index of plasma with prediabetes and type 2 diabetes mellitus among Chinese adults aged 45 years and above: a cross-sectional study from CHARLS. Front Endocrinol. (2024) 15:1360874. doi: 10.3389/fendo.2024.1360874 PMC11018972 38628590

[33] Kammar-García A López-Moreno P Hernández-Hernández ME Ortíz-Bueno AM Martínez-Montaño MLC. Atherogenic index of plasma as a marker of cardiovascular risk factors in Mexicans aged 18 to 22 years. Proc Bayl Univ Med Cent. (2020) 34. doi: 10.1080/08998280.2020.1799479 PMC7785179 33456139

[34] Zhou K Qin Z Tian J Cui K Yan Y Lyu S. The atherogenic index of plasma: A powerful and reliable predictor for coronary artery disease in patients with type 2 diabetes. Angiology. (2021) 72. doi: 10.1177/00033197211012129 33949211

[35] Płaczkowska S Sołkiewicz K Bednarz-Misa I Kratz EM. Atherogenic plasma index or non-high-density lipoproteins as markers best reflecting age-related high concentrations of small dense low-density lipoproteins. Int J Mol Sci. (2022) 23:5089. doi: 10.3390/ijms23095089 35563477 PMC9102874

[36] Ichikawa T Okada H Hamaguchi M Kurogi K Murata H Ito M. Estimated small dense low-density lipoprotein-cholesterol and incident type 2 diabetes in Japanese people: Population-based Panasonic cohort study 13. Diabetes Res Clin Pract. (2023) 199. doi: 10.1016/j.diabres.2023.110665 37031889

[37] Hirano T. Pathophysiology of diabetic dyslipidemia. J Atheroscler Thromb. (2018) 25:771–82. doi: 10.5551/jat.RV17023 PMC6143775 29998913

[38] Bersch-Ferreira ÂC Stein E Waclawovsky G da Silva LR Machado RHV Weschenfelder C. Effect of nuts on lipid profile and inflammatory biomarkers in atherosclerotic cardiovascular disease: a systematic review and meta-analysis of randomized controlled trials. Eur J Nutr. (2024) 63. doi: 10.1007/s00394-024-03455-2 38967674

[39] Haffner SM MykkÄnen L Robbins D Valdez R Miettinen H Howard BV. A preponderance of small dense LDL is associated with specific insulin, proinsulin and the components of the insulin resistance syndrome in non-diabetic subjects. Diabetologia. (1995) 38:1328–36. doi: 10.1007/BF00401766 8582543

[40] Pradhan AD Ridker PM. Do atherosclerosis and type 2 diabetes share a common inflammatory basis? Eur Heart J. (2002) 23. doi: 10.1053/euhj.2001.3052 12042000

[41] Krakoff J Funahashi T Stehouwer CD Schalkwijk CG Tanaka S Matsuzawa Y. Inflammatory markers, adiponectin, and risk of type 2 diabetes in the Pima Indian. Diabetes Care. (2003) 26. doi: 10.2337/diacare.26.6.1745 12766104

[42] Sjöholm A Nyström T. Inflammation and the etiology of type 2 diabetes. Diabetes Metab Res Rev. (2006) 22. doi: 10.1002/dmrr.568 15991254

[43] Hannuksela ML Liisanantti MK Savolainen MJ. Effect of alcohol on lipids and lipoproteins in relation to atherosclerosis. Crit Rev Clin Lab Sci. (2002) 39. doi: 10.1080/10408360290795529 12120782

