# Supplementary material for: Association between the plasma atherogenic index and type 2 diabetes in Chinese population: prospective cohort study based on 4C study
Source: Front Endocrinol (Lausanne). 2025 Apr 16;16:1571602. doi: 10.3389/fendo.2025.1571602 (PMC12040675; doi:10.3389/fendo.2025.1571602)
Supplement: Supplementary file 1 [file DataSheet1.docx]

**Supplementary material**

| **Table S1 Baseline characteristics of participants by follow-up situation** | | | |  |
| --- | --- | --- | --- | --- |
| **Variables** | **Non-missing respondents** | **Missing respondents** | **P** |  |
| N | 9092 | 882 |  |  |
| Age, years | 58.50±10.02 | 58.40±10.00 | 0.73 |  |
| Male (%) | 3085(33.9) | 297(33.7) | 0.56 |  |
| BMI, kg/m^2^ | 23.91±3.35 | 23.93±3.39 | 0.32 |  |
| DBP, mmHg | 77.62±11.54 | 78.26±12.00 | 0.89 |  |
| SBP, mmHg | 127.40±20.92 | 127.38±20.79 | 0.28 |  |
| LDL-c, mmol/L | 2.59±0.82 | 2.61±0.87 | 0.53 |  |
| HDL-c, mmol/L | 1.24±0.34 | 1.26±0.35 | 0.42 |  |
| TC, mmol/L | 1.62±1.28 | 1.63±1.29 | 0.54 |  |
| TG, mmol/L | 4.59±1.13 | 4.63±1.16 | 0.18 |  |
| Cr, mmol/L | 65.10±21.80 | 64.39±18.09 | 0.21 |  |
| FBG, mmol/L | 5.92±1.68 | 5.94±1.65 | 0.51 |  |
| HbA1c, % | 6.16±1.10 | 6.13±1.11 | 0.90 |  |
| AIP | 0.05±0.29 | 0.04±0.30 | 0.19 |  |
| Current smoker | 1308(14.4) | 122(13.8) | 0.29 |  |
| Current drinker | 2515(27.7) | 234(26.5) | 0.38 |  |
| Senior high school and above | 2689(29.5) | 262(29.7) | 0.96 |  |
| Data are summarized as number (percentage), mean ± standard deviation. AIP, plasma atherogenic index; BMI, body mass index; DBP, diastolic blood pressure; SBP, systolic blood pressure; LDL-C, low-density lipoprotein cholesterol; HDL-C, high-density lipoprotein cholesterol; TC, total cholesterol; TG, triglyceride; Cr, creatinine; FBG, fasting blood glucose; HbA1c, glycated haemoglobin. *P<0.05 | | | |  |


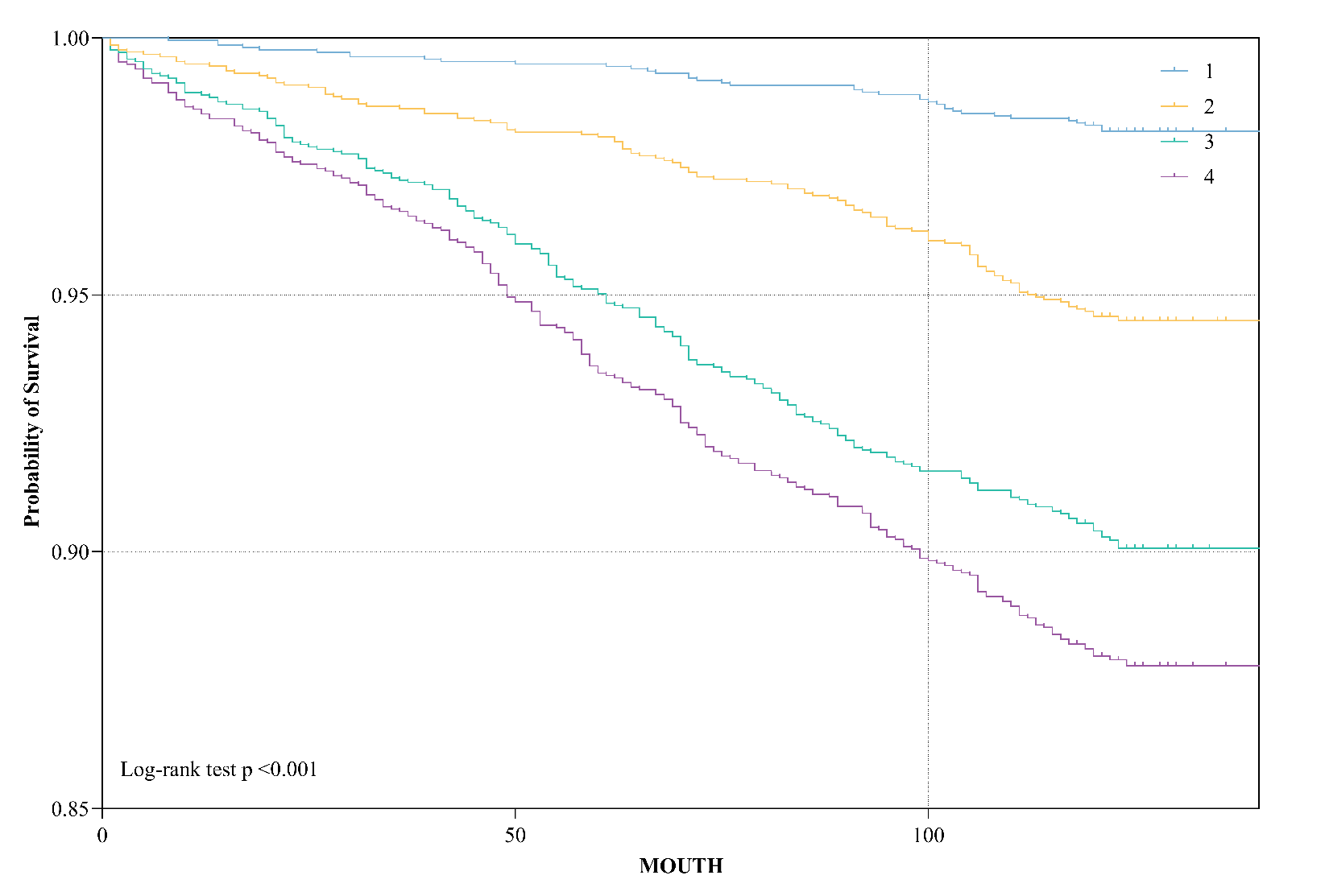


Figure S1. Kaplan-Meier curves for cumulative incidence of type 2 diabetes.


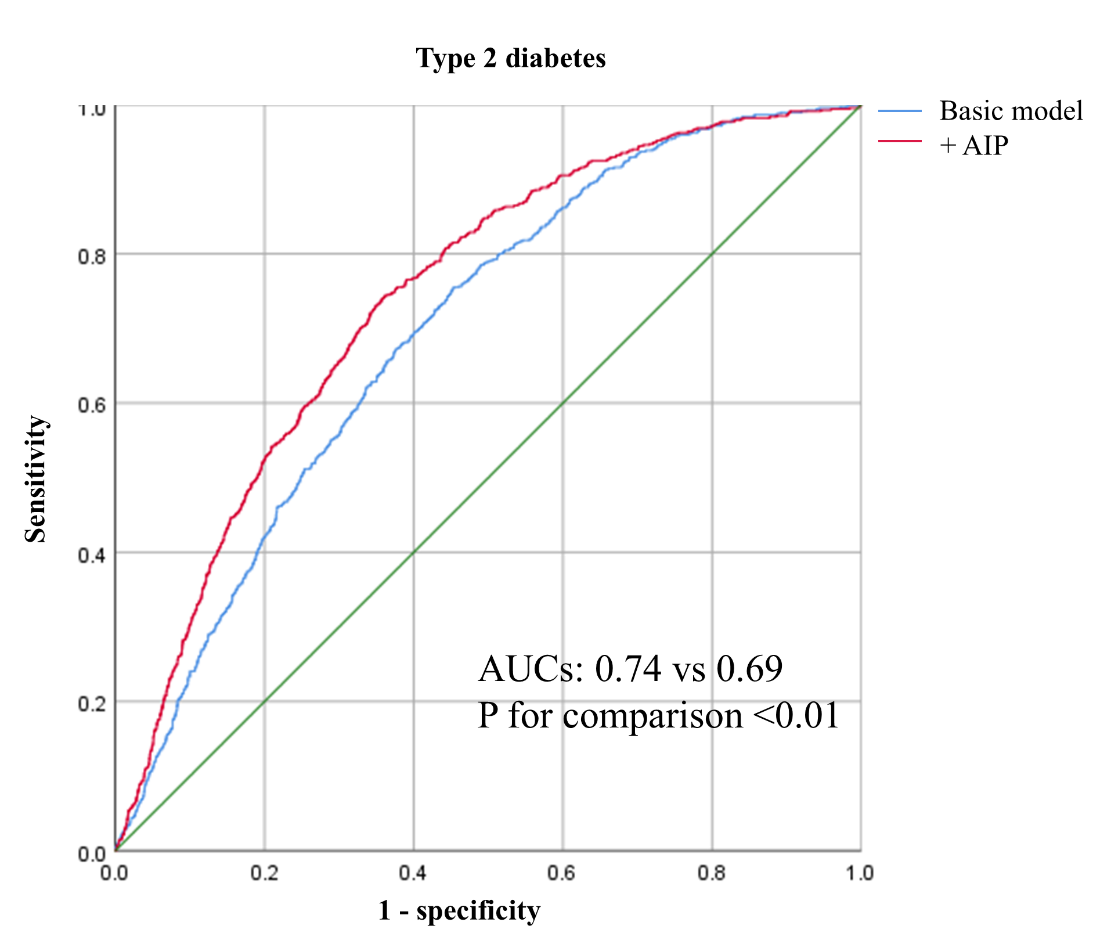


Figure S2. ROC curves for AIP prediction of type 2 diabetes. The base model was adjusted for age, sex, BMI, DBP, SBP, HDL-C, LDL-C, TG, TC, Cr, FBG, HbA1c, smoking status, alcohol consumption, and education level.
